# SIRT1 Inhibits Apoptosis by Promoting Autophagic Flux in Human Nucleus Pulposus Cells in the Key Stage of Degeneration via ERK Signal Pathway

**DOI:** 10.1155/2021/8818713

**Published:** 2021-02-27

**Authors:** Fei He, Qingshu Li, Bo Sheng, Haitao Yang, Wei Jiang

**Affiliations:** ^1^Department of Orthopedics, The First Affiliated Hospital of Chongqing Medical University, Chongqing 400016, China; ^2^Department of Pathology, Chongqing Medical University, Chongqing 400010, China; ^3^Department of Radiology, The First Affiliated Hospital of Chongqing Medical University, Chongqing 400016, China

## Abstract

**Background:**

The application of biomolecular interventions in the early stage of intervertebral disc degeneration (IVDD) is considered an ideal method for the treatment of IVDD. However, the precise definition of the “early stage” of IVDD is unclear. Silent information regulation 2 homologue-1 (SIRT1) can protect human degenerative nucleus pulposus (NP) cells from apoptosis by activating autophagy. However, the mechanism of this effect is still unclear. This study tried to confirm the “early stage” of IVDD and the role of NP cell autophagy during IVDD as well as to determine the mechanism by which SIRT1 protects NP cells.

**Methods:**

The characteristics of the NP in various stages of degeneration were assessed to confirm the “early stage” of IVDD. Then, autophagy and apoptosis were detected in NP cells after SIRT1 upregulation/downregulation. Finally, LY294002 and PD98059 were used to inhibit the AKT/ERK pathway to determine the mechanism by which SIRT1 regulates autophagy in NP cells.

**Results:**

Our data showed that mildly degenerative (Pfirrmann grade III with normal height of intervertebral disc) NP cells may be the key target for biomolecular interventions in IVDD and that SIRT1 protects human mildly degenerative NP cells from apoptosis by activating autophagy via the ERK signalling pathway.

**Conclusion:**

Our data showed that SIRT1 inhibits apoptosis by promoting the autophagic flux in NP cells via the ERK signalling pathway during the key stage of degeneration. These findings will assist in the development of novel therapeutic approaches for IVDD treatment.

## 1. Introduction

Intervertebral disc degeneration (IVDD) is the main cause of low back pain (LBP) [1, 2] and the pathological basis of spinal degenerative diseases [3, 4]. Biological therapies with promising treatment modalities for IVDD can impact the future management of LBP. Inhibition or reversion of IVDD in the early stage with biomolecular interventions is considered a better choice [5, 6]. However, the precise definition of “early stage” of IVDD is unclear.

The molecular mechanisms involved in the pathogenesis of IVDD are unclear; however, many studies, including our previous reports, have demonstrated that excessive apoptosis of nucleus pulposus (NP) cells is one of the major cellular and biochemical changes in the degenerative discs [7–10]. Excessive apoptosis of NP cells leads to the degradation of NP extracellular matrix (ECM), which supports the mechanical function of the discs. Thus, inhibition of excessive apoptosis of NP cells may decrease the degradation of ECM and postpone the progression of IVDD.

Autophagy maintains the survival of cells under stress, such as ischaemia and hypoxia, by scavenging senescent organelles and misfolded proteins [11, 12]. In the case of persistent stress, autophagy fails to support cell survival, and apoptosis is activated to avoid local inflammatory reactions and remove debris generated by cell lysis [13, 14]. Substantial evidence has confirmed that autophagy effectively protects cell survival by inhibiting apoptosis [15–18]; we have also confirmed that autophagy can protect against apoptosis in degenerative human NP cells [19]. However, the activity of autophagy in the process of IVDD is a matter of controversy. Ye et al. [20, 21] reported that autophagy was increased during the pathological process of IVDD and ageing in rat NP cells. However, our early study demonstrated that autophagy of NP cells was lower in severely degenerative discs than that in normal discs [19]. Determination of the specific roles of autophagy in the process of IVDD, especially in humans, is required for the biomolecular treatment of IVDD.

Silent information regulation 2 homologue-1 (SIRT1) is an NAD-dependent deacetylase that plays the key roles in multiple cellular processes, including cell cycle, metabolism, autophagy, and apoptosis [22–24]. Our previous studies demonstrated that the expression of SIRT1 is decreased in IVDD [10, 19, 25]. Furthermore, we confirmed that resveratrol, a natural agonist of SIRT1, protects against apoptosis by promoting autophagy in human NP cells from mild degenerative discs [19]. However, resveratrol is not a specific activator of SIRT1 and may also activate other sirtuin enzymes to exert other biological effects [26]. To verify the exact mechanism of action of SIRT1 in NP cells, lentiviral vectors overexpressing SIRT1 (LV-SIRT1) and small interfering RNAs (siRNAs) for silencing SIRT1 were used to target and regulate SIRT1 expression in the present study.

Phosphatidylinositol 3-kinase (PI3K)/AKT is the main intracellular signalling pathway that has been reported to induce autophagy in several cell types [27–29]. Recently, Maito et al. [30] demonstrated that activation of the AKT pathway protects against apoptosis and induces autophagy in human disc NP cells. To date, there are no reports on the molecular mechanism by which the SIRT1-AKT pathway regulates autophagy in human disc NP cells. The extracellular signal-regulated kinase (ERK) pathway was also reported to regulate apoptosis, proliferation, and differentiation of the cells [31]. However, it is unclear whether the SIRT1-ERK pathway regulates autophagy in human disc NP cells. In this study, we redefined the “early stage” of IVDD with regards to biomolecular interventions and investigated specific roles of autophagy in the process of human IVDD. We also demonstrated that SIRT1 protects against apoptosis by promoting autophagy and determined the mechanism by which SIRT1 regulates the autophagic flux in human mildly degenerative NP cells.

## 2. Materials and Methods

### 2.1. Patients and NP Tissue Samples

The degeneration grade of IVDD was classified according to the Pfirrmann classification by a magnetic resonance imaging (MRI) scan of the spine prior to surgery [32]. Specifically, grade III was differentiated into two phases depending on whether the height of intervertebral disc (IVD) was normal. Normal NP tissues (grade I) were obtained from 5 patients with lumbar vertebral fracture (LVF) without documented clinical history of LBP (3 women and 2 men; mean age, 32.30 ± 6.83 years). Mild degenerative NP tissues (grade III with normal height of IVD) were obtained from 8 patients (3 women and 5 men; mean age, 34.56 ± 5.93 years), and severe degenerative NP tissues (grade V) were obtained from 4 patients (2 women and 2 men; mean age, 35.26 ± 6.48 years) with degenerative disc disease (DDD). The study was compliant with the Declaration of Helsinki and was approved by the Ethics Committee of Chongqing Medical University; an informed consent was obtained from all patients enrolled in the study.

### 2.2. MRI Scanning and Quantitative Analysis

The lumbar spine was imaged using a 1.5 T System (Siemens, Germany) with a surface coil. Midsagittal T2-weighted MRIs were used for the evaluation of the discs. The signal intensity of the discs on the images was analysed by Vue PACS (Virtual Reading Profile, Carestream Health, Inc.). Multiplanar virtual reading (MPVR) was used to reconstruct three-dimensional (3D) colour MRI images.

### 2.3. Chemicals and Antibodies

Dulbecco's modified Eagle's medium and Ham's F-12 medium (DMEM/F12, 1 : 1, Gibco, USA), foetal bovine serum (FBS; Gibco, USA), rabbit anti-SIRT1 antibodies (Epitomics, USA), rabbit anti-collagen II antibodies, rabbit anti-aggrecan antibodies, rabbit anti-LC3 antibodies, rabbit anti-p62 antibodies, rabbit anti-AKT antibodies, rabbit anti-p-AKT antibodies, rabbit anti-ERK1/2 antibodies, rabbit-p-ERK1/2 antibodies, LY294002, PD98059, chloroquine (CQ; Sigma-Aldrich, USA), mouse anti-*β*-actin antibodies, goat anti-rabbit IgG-HRP antibodies, and goat anti-mouse IgG-HRP antibodies (Beyotime, China).

### 2.4. Cell Culture

Mild degenerative NP tissues (grade III with normal height of IVD) were washed twice with phosphate-buffered saline (PBS) and cut into 1 mm^3^ fragments within 30 min after being harvested. Then, the tissue fragments were digested in 0.25% trypsin solution for 30 min and in 0.2% type II collagenase for 3-4 h at 37°C. Then, NP cells were filtered and cultured as a monolayer in DMEM/F12 supplemented with 15% FBS and 1% penicillin-streptomycin as described previously [19]. To maintain the NP phenotype, the cells were cultured in a three-dimensional (3D) cell culture model by encapsulating in Alvetex® (Reinnervate, Durham, UK) as reported [33] after the first passage. Briefly, the inserts were submerged in 70% ethanol for 10 min, washed twice with sterile water, incubated with poly-L-ornithine (1.5 *μ*g/mL) for 24 h, washed with PBS, and finally incubated in DMEM/F12 with 15% FBS and 1% penicillin-streptomycin for 2 h at 37°C in an atmosphere of 5% CO_2_. After the first passage, NP cells were seeded into 6-well plates containing Alvetex® inserts at a density of 1 × 10^6^ cells/mL.

### 2.5. Lentiviral Vector Transduction and siRNA Transfection

NP cell monolayer was transduced with the lentiviral vector or transfected with siRNAs before seeding into the Alvetex® inserts. Recombinant LV-SIRT1 was purchased from GeneChem Co., Ltd. (Shanghai, China). NP cells were seeded in 6-well plates for 24 hours and transduced with 5 *μ*L of LV-SIRT1 or negative control lentiviral vector (NC-LV) by using polybrene (8 *μ*g/mL). NP cells were seeded into 6-well plates containing the Alvetex® inserts 72 hours after the transduction. Double-stranded small interfering RNA (siRNA) for human SIRT1 gene silencing was purchased from Invitrogen Co., Ltd. (USA). The cells were seeded in 6-well plates for 24 h and transfected with SIRT1 or negative control siRNA duplexes using PepMute siRNA transfection reagent (SignaGen, USA) according to the manufacturer's instructions. After 72 hours of transfection, NP cells were seeded into 6-well plates containing the Alvetex® inserts.

### 2.6. Detection of Apoptosis by Flow Cytometry

An Annexin V/PI double-staining flow cytometry kit (Keygen, China) was used to detect apoptosis of NP cells. NP cells were harvested after various treatments and washed twice with PBS. Then, NP cells were incubated in binding buffer containing Annexin-PE and PI in the dark for 10-15 min at room temperature. Then, flow cytometry was performed within 1 h. The cells with positive staining for Annexin V+/PI- and Annexin V+/PI+ were counted, and the data are presented as the percentage of total cell count.

### 2.7. Western Blot Analysis

The NP samples from 3 LVF patients (grade I, total number of LVF patients was 4), 3 DDD patients (grade III with normal height of IVD, total number of patients was 8), and 3 DDD patients (grade V, total number of patients was 4) were randomly selected for western blot analysis. Protein was extracted using a tissue protein extraction kit (ComWin Biotechnology, CW0891) according to the manufacturer's instructions. NP cells from mild degenerative NP tissues (grade III) were harvested, washed in ice-cold PBS (pH = 7.5), and lysed using RIPA lysis buffer (Beyotime, P0013B). Samples from the tissues and cells containing 50 *μ*g proteins were separated by sodium dodecyl sulfate- (SDS-) polyacrylamide gel electrophoresis (PAGE) through a 6%~12% gel, and the proteins were transferred to 0.45 *μ*m or 0.22 *μ*m polyvinylidene difluoride (PVDF) membranes. The membranes were incubated with rabbit anti-collagen II (1 : 100), rabbit anti-aggrecan (1 : 100), rabbit anti-SIRT1 (1 : 2,000), rabbit anti-LC3 (1 : 1,000), rabbit anti-p62 (1 : 1,000), rabbit anti-AKT (1 : 1,000), rabbit anti-p-AKT (1 : 1,000), mouse anti-beclin-1 (1 : 1,000), rabbit anti-ERK1/2 (1 : 1,000), rabbit-p-ERK1/2 (1 : 1,000), and mouse anti-*β*-actin (1 : 500) primary antibodies overnight at 4°C. Then, the membranes were washed and incubated with goat anti-rabbit IgG-HRP (1 : 5,000) or goat anti-mouse IgG-HRP (1 : 5,000) secondary antibodies for 1 h at 37°C. The membranes were washed, and the proteins were visualized with ECL Plus reagent (Invitrogen, WP20005) using a ChemiDoc XRS + imaging system (Bio-Rad, USA).

### 2.8. Statistical Analysis

Data are expressed as the mean ± standard deviation of three independent experiments. Statistical differences were estimated using Student's *t*-test for comparison between two groups or analysis of variance (ANOVA) followed by Tukey's test for comparison of multiple groups. The SPSS 14.0 statistical software (SPSS Inc., IL, USA) was used for statistical analyses, and *P* < 0.05 was considered statistically significant.

## 3. Results

### 3.1. Pfirrmann Grade III with Normal Height Is the Key Stage of IVDD

**Figure 1:**
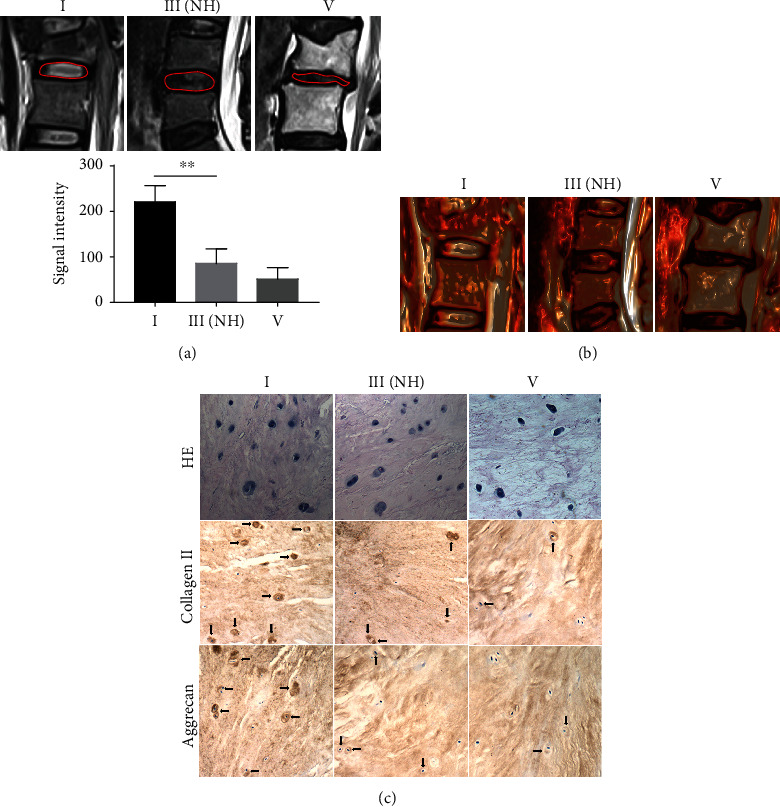
Pfirrmann grade III with normal height (NH) is the key stage of IVDD with the highest autophagy in NP cells. (a) Representative plain scanning and NP signal intensity of MRI of grades I, III(NH), and V according to the Pfirrmann classification. (b) The reconstructed 3D colour images of various stages of IVDD. (c) HE staining and immunohistochemistry analysis of collagen II and aggrecan in human NP tissues from the grade I, III(NH), and V groups. Arrows indicate positive cells. The values are shown as the mean ± standard deviation (S.D.) of three independent experiments performed in triplicate. ^∗^*P* < 0.05, ^∗∗^*P* < 0.01. NH: normal height.

Multiple approaches, including qualitative and quantitative analysis of MRI and haematoxylin and eosin (HE) and immunohistochemical (collagen II and aggrecan) staining, were used to determine the key stage of IVDD for biomolecular interventions based on the characteristics of the NP in various stages of degeneration.  Figure 1(a) shows the MRI data of various degenerative IVDD stages according to the Pfirrmann classification. Specifically, mild degenerative IVDD was grade III with a normal height of IVD. The signal intensity of NP was reduced in grade III with a normal height of IVD compared with that in stage I and was the lowest in grade V. The reconstructed 3D colour images confirmed the results (Figure 1(b)). The results of HE staining showed that the number of NP cells was visually reduced in severely degenerative NP tissues compared with that in the normal and mildly degenerative NP tissues (Figure 1(c)). The results of immunohistochemical staining for collagen II indicated that the number of positive cells was reduced, and the intensity of the signal was decreased in mildly degenerative NP cells compared with those in normal NP cells; the number of positive cells was the lowest and the signal intensity was the weakest in severe degenerative NP (Figure 1(c)). The pattern of aggrecan expression was similar to that of collagen II expression in various degenerative NP tissues (Figure 1(c)). These results showed that NP cells in grade III IVDD with a normal height of IVD were similar to those in the normal intervertebral discs; however, the ability of NP cells to secrete ECM was decreased. Thus, inhibition or reversal of IVDD in this stage by increasing the ability of NP cells to secrete ECM may be achieved by biomolecular interventions.

### 3.2. The Activity of Autophagy in NP Cells Is the Highest in Mildly Degenerative Discs in Patients

**Figure 2:**
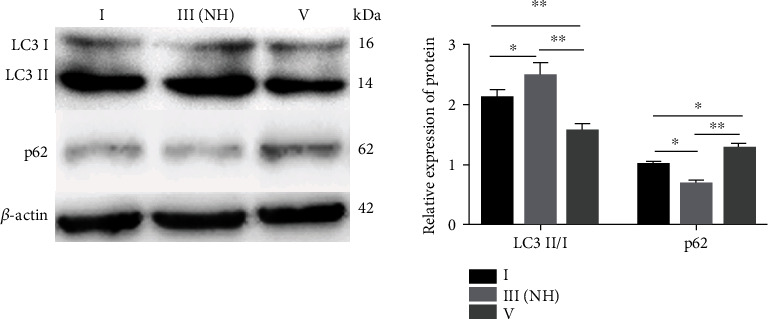
Western blot analysis of LC3II/I and p62 expression in NP cells from the grade I, III(NH), and V groups. The relative protein expression of LC3II/I corresponds to the ratio of LC3II signal to LC3I signal, and p62 corresponds to the ratio of p62 signal to *β*-actin signal. The values are shown as the mean ± standard deviation (S.D.) of three independent experiments performed in triplicate. ^∗^*P* < 0.05, ^∗∗^*P* < 0.01. NH: normal height.

To confirm the relationship between the activity of autophagy in NP cells and IVDD, we performed western blot to detect the expression of LC3 and p62 in normal, mildly degenerative (Pfirrmann grade III with normal height of IVD) and severely degenerative (Pfirrmann grade V) NP. The results showed that the expression of LC3II/I protein was the highest in mild degenerative NP and was lower in severe degenerative NP than that in normal NP; the p62 protein expression was the lowest in mild degenerative NP and higher in severe degenerative NP than that in normal NP (Figure 2). These results indicated that the activity of autophagy in NP cells is the highest in mild degenerative discs in patients.

### 3.3. SIRT1 Enhances Autophagic Flux and Inhibits Apoptosis in Mildly Degenerative NP Cells

**Figure 3:**
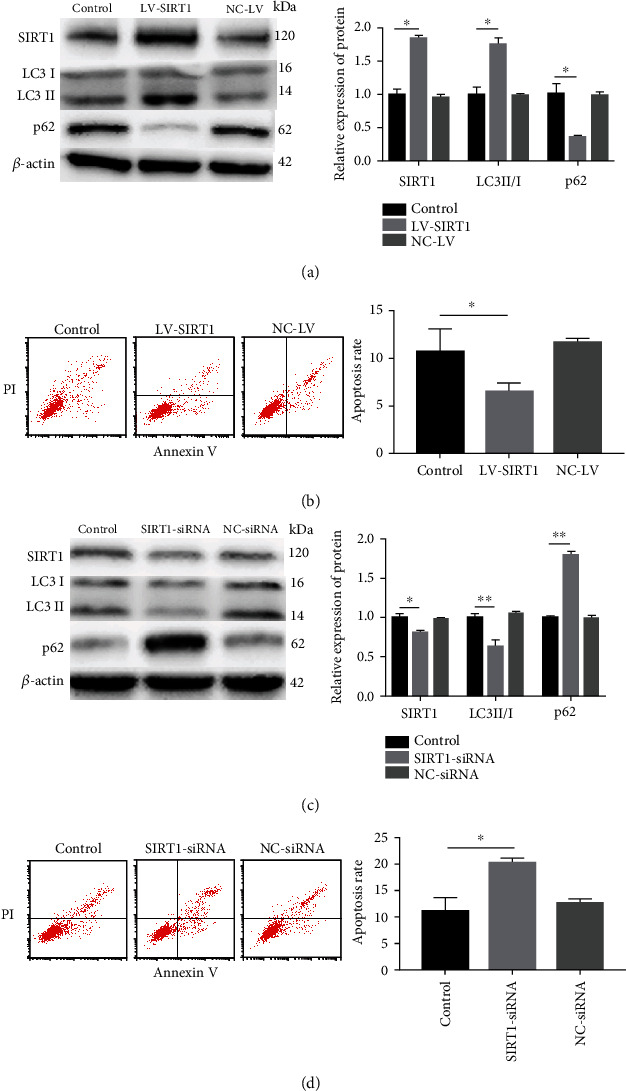
Overexpression or downregulation of SIRT1 enhances or inhibits autophagic flux and inhibits or increases apoptosis, respectively, in mild degenerative NP cells. (a) The levels of SIRT1 and autophagy-related proteins were detected by western blot. SIRT1 expression was significantly elevated in mild degenerative human NP cells transfected with LV-SIRT1. LC3II/I level was elevated and p62 level was significantly reduced after LV-SIRT1 transfection. (b) The apoptosis rate of mildly degenerative human NP cells was evaluated by flow cytometry. The apoptosis rate was significantly decreased in NP cells transfected with LV-SIRT1. (c) The levels of SIRT1 and autophagy-related proteins were detected by western blot. SIRT1 expression was significantly reduced in mild degenerative human NP cells treated with SIRT1-siRNA. LC3II/I level was reduced and p62 level was increased after SIRT1-siRNA treatment. (d) The apoptosis rate of mildly degenerative human NP cells was evaluated by flow cytometry. The apoptosis rate was increased in NP cells treated with SIRT1-siRNA. The relative protein expression of LC3II/I corresponds to the ratio of LC3II signal to LC3I signal; p62 and SIRT1 correspond to the ratio of p62 and SIRT1 signals, respectively, to *β*-actin signal. The values are shown as the mean ± standard deviation (S.D.) of three independent experiments performed in triplicate. ^∗^*P* < 0.05, ^∗∗^*P* < 0.01.

To clarify the effects of SIRT1 on autophagic flux and apoptosis of mildly degenerative NP cells, we used LV-SIRT1 or SIRT1-siRNA to promote or inhibit SIRT1 expression, respectively. Western blot was used to detect the expression of the key autophagic flux proteins and SIRT1. Flow cytometry was used to detect apoptosis of NP cells. Western blot analysis confirmed that transduction or transfection of NP cells with LV-SIRT1 or SIRT1-siRNA effectively increased or inhibited SIRT1 protein expression, respectively, compared with that in control NP cells (Figures 3(a) and 3(c)). Overexpression of SIRT1 induced an increase in the LC3II/I ratio and a decrease in the p62 protein level compared with those in control NP cells (Figure 3(a)), and the apoptosis rate of NP cells was significantly reduced after SIRT1 overexpression (Figure 3(b)). Knockdown of SIRT1 induced a decrease in the LC3II/I ratio and an increase in the p62 level (Figure 3(c)), and the apoptosis rate was significantly increased after SIRT1 knockdown (Figure 3(d)). These results indicated that SIRT1 promotes autophagic flux and inhibits apoptosis in mildly degenerative NP cells.

### 3.4. SIRT1 Inhibits Apoptosis of Mildly Degenerative NP Cells by Enhancing Autophagic Flux

**Figure 4:**
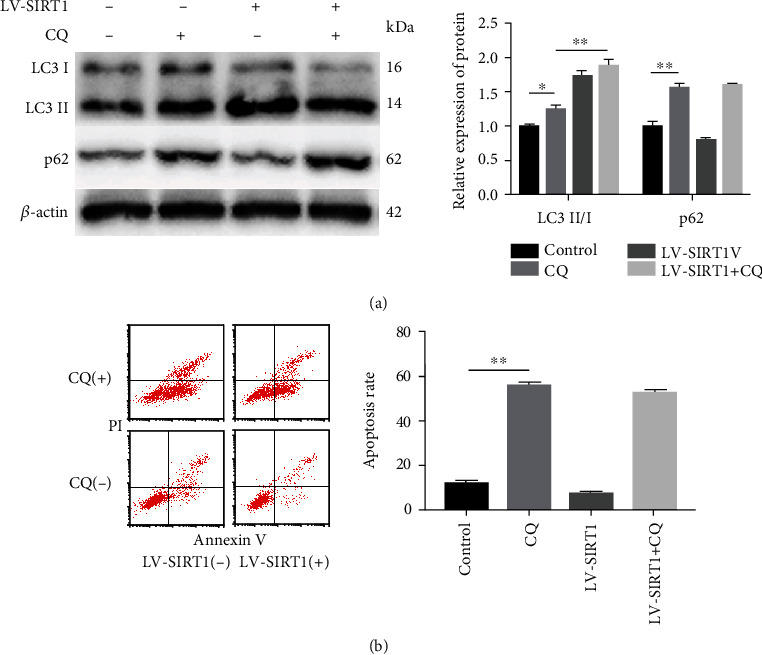
SIRT1 inhibits apoptosis of mild degenerative NP cells by enhancing autophagic flux. (a) LC3II/I and p62 protein levels were detected by western blot. The expression of LC3II/I and p62 was significantly increased in mildly degenerative human NP cells treated with CQ (40 *μ*M) in combination with LV-SIRT1 transfection. (b) The apoptosis rate of mildly degenerative human NP cells evaluated by flow cytometry. The apoptosis rate was significantly increased in NP cells treated with CQ (40 *μ*M), and there were no significant differences between NP cells treated with CQ+LV-SIRT1 and NP cells treated with CQ (40 *μ*M). The relative protein expression of LC3II/I corresponds to the ratio of LC3II signal to LC3I signal, and p62 corresponds to the ratio of p62 signal to *β*-actin signal. The values are shown as the mean ± standard deviation (S.D.) of three independent experiments performed in triplicate. ^∗^*P* < 0.05, ^∗∗^*P* < 0.01.

To determine whether SIRT1 inhibits apoptosis of mildly degenerative NP cells by enhancing autophagic flux, the autophagy inhibitor chloroquine (CQ) was used to inhibit the degradation of the autophagic lysosome. Western blot analysis confirmed that treatment with CQ (40 *μ*M) significantly increased the expression of p62 and the LC3II/I ratio. SIRT1 overexpression combined with CQ increased the LC3II/I ratio compared with that in cells treated with CQ alone; however, the p62 level was not significantly changed (Figure 4(a)). The results of flow cytometry showed that CQ increased the apoptosis rate of NP cells. However, overexpression of SIRT1 did not reverse an increase in apoptosis caused by CQ pretreatment (Figure 4(b)). These results indicated that SIRT1 inhibits apoptosis by enhancing autophagic flux in mildly degenerative NP cells.

### 3.5. SIRT1 Promotes Autophagy via the ERK, but Not AKT Pathway in Mildly Degenerative NP Cells

**Figure 5:**
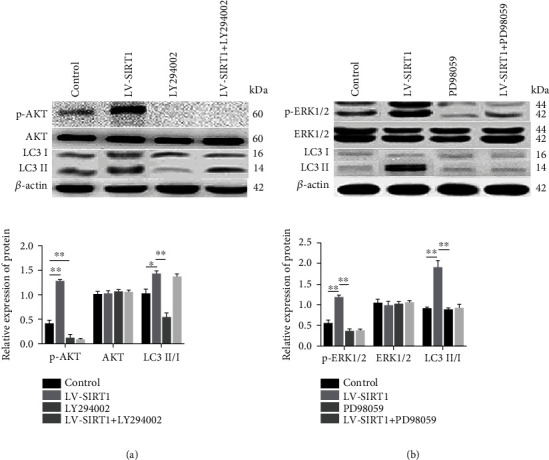
SIRT1 promotes autophagy via the ERK, but not AKT, pathway in mildly degenerative NP cells. (a) The levels of p-AKT, AKT, and LC3 II/I proteins were detected by western blot. LY294002 inhibited autophagy (LC3II/I was decreased) by inhibiting p-AKT; however, LV-SIRT1 partly reversed the effect of LY29004002 and promoted autophagy (an increase in LC3II/I expression). (b) The levels of p-ERK1/2, ERK1/2, and LC3II/I proteins were detected by western blot. PD98059 inhibited autophagy (LC3II/I was decreased) by inhibiting p-ERK1/2, and LC3II/I expression did not change after the treatment with LV-SIRT1 combined with PD98059. The relative protein expression of LC3II/I corresponds to the ratio of LC3II signal to LC3I signal, and p-AKT and AKT correspond to the ratio of p-AKT and AKT signals, respectively, to *β*-actin signal. The values are shown as the mean ± standard deviation (S.D.) of three independent experiments performed in triplicate.

**Figure 6:**
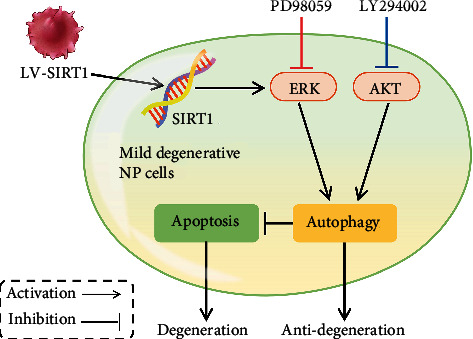
Schematic representation of the proposed mechanism by which SIRT1 inhibits apoptosis by promoting autophagic flux through the ERK, but not AKT, pathway in mildly degenerative human intervertebral disc NP cells.

We investigated whether the AKT and/or ERK pathways are involved in the regulatory effects of SIRT1 on autophagy in mild degenerative NP cells. The PI3K inhibitor LY294002 was used to inhibit AKT, and NP cells were transfected with LV-SIRT1 to determine whether overexpression of SIRT1 can influence this effect. The results of western blot showed that LY294002 almost completely blocked the phosphorylation of AKT (p-AKT), and LV-SIRT1 activated p-AKT. However, when the level of p-AKT was inhibited by LY294002, LV-SIRT1 did not increase the expression of p-AKT and promoted autophagy (an increase in the LC3II/I ratio) (Figure 5(a)). These results indicated that SIRT1 may regulate the autophagy pathway via a mechanism independent of PI3K/AKT in mild degenerative NP cells (Figure 6).

The ERK inhibitor PD98059 was used to inhibit ERK, and NP cells were transfected with LV-SIRT1 to determine whether overexpression of SIRT1 can influence this effect. The results of western blot showed that PD98059 effectively blocked the phosphorylation of ERK1/2 (p-ERK1/2) and decreased the LC3II/I ratio. Overexpression of SIRT1 alone activated p-ERK1/2; however, when p-ERK1/2 was inhibited by PD98059, LV-SIRT1 did not increase the LC3II/I ratio (Figure 5(b)). These results indicated that SIRT1 promotes autophagy via the ERK pathway in human mild degenerative NP cells (Figure 6).

## 4. Discussion

Biological therapies are a promising treatment modality for DDD that can impact future management of LBP [5]. Many biological therapies, including protein injections [34], gene-based therapy [35], cell therapy [36], and tissue engineering [37], have been applied to treat DDD. The therapeutic mode depends on the degree of degeneration of IVDD: in the mild degenerative stage, biomolecular interventions can rebalance the anabolic and catabolic pathways in the degenerative cascade; in the intermediate degenerative stage, cell implantation can be applied to repopulate the disk; and in the severe degenerative stage, tissue engineering constructs have to be employed for biological reconstruction [5, 6]. Inhibition or reversion of IVDD in the mild degenerative stage with better biomolecular interventions can avoid subsequent more complex treatments. However, there is no strict definition of the “mild degenerative stage.” According to the Pfirrmann grading system, grades I and II have normal IVD [38]. We hypothesise that Pfirrmann grade III with normal height is mild degenerative IVDD. The results of quantitative MRI showed that the NP signal intensity in the case of Pfirrmann grade III with normal height was reduced compared with that in normal IVD, indicating that the water content is reduced at this stage. However, the normal height of IVD means that the main structure of IVD has not been damaged. The results of HE staining showed that the number of NP cells in mild degenerative IVDD was as high as that in normal IVD; however, the expression of collagen II and aggrecan in NP cells in mild degenerative IVDD was decreased compared to those in normal IVD. These results indicated that Pfirrmann grade III with normal height is the early stage of IVDD and that biomolecular interventions may be a preferred method for the inhibition or reversal of IVDD [5, 6].

Autophagy is a homeostatic mechanism that eliminates protein aggregates and damaged organelles in response to increased metabolic demands or stresses and has been shown to be a promising target for mitigating IVDD [39]. The specific role of autophagy in IVDD is controversial. Ye et al. [20, 21] reported that autophagy was increased in rat NP in the pathological process of IVDD and during ageing. However, our previous study demonstrated that the activity of autophagy in NP cells was decreased in severely degenerative discs compared to that in normal discs [19]. To solve this controversy in the present study, the activity of autophagic flux was detected in NP cells in patients with normal IVD and in mild and severe degenerative IVDD. The results confirmed that the activity of autophagy in NP cells is the highest in mild human degenerative IVDD and is decreased in severe degenerative IVDD compared to that in normal IVD. These results indicate that autophagy is involved in the maintenance of the integrity and survival of normal NP cells [40]. As degeneration progresses, the activity of autophagic flux is increased to protect the cells. In the case of persistent stress, autophagy fails to support cell survival resulting in the activation of apoptosis to avoid local inflammatory reactions and remove debris generated by cell lysis; finally, the activity of autophagy decreases in the severe degenerative stage. Comparison of human IVDD progression indicates that ageing or severe degenerative IVDD in rats may be equivalent to the mild stage because of the characteristics of rats that lack bipedalism [41]. Hence, our current findings may explain previous controversial results about the role of autophagy in IVDD in rat and human studies. Our results indicated that Pfirrmann grade III with a normal height of IVD may be the key stage for autophagy-related interventions in NP cells in IVDD progression. Therefore, NP cells in this stage were selected for further study. Our study is the first to limit the investigation to experimental interventions in NP cells.

SIRT1 has been reported to play a protective role in IVDD [8, 19, 25]. Our previous study confirmed that resveratrol increases the expression of SIRT1 and inhibits apoptosis of degenerative NP cells by promoting autophagy in in vitro monolayer culture [19]. However, the results of a recent study challenged the general use of resveratrol as a pharmacological tool for activating SIRT1 [26]. Therefore, we targeted the regulation of SIRT1 by lentivirus transduction and siRNA transfection in NP cells. Moreover, considering phenotypic changes in NP cells in the monolayer culture, we used 3D culture of NP cells. Our results indicated that SIRT1 protects mildly degenerative NP cells against apoptosis by increasing autophagic flux. This result is consistent with our previous reports and other results on the protective role of SIRT1 in IVDD. Only NP cells from Pfirrmann grade III IVDD were selected in this study, and Pfirrmann grade III is the key stage for biomolecular therapy; thus, these results are important for further clinical treatment.

The activated PI3K/AKT signalling pathway has been confirmed to induce autophagy in several cell types [27, 42]. In the present study, LY294002 effectively blocked p-AKT protein expression, and LV-SIRT1 activated p-AKT. Furthermore, we observed that LV-SIRT1 failed to override the inhibitory effect of LY294002 on p-AKT but effectively increased LC3II/I expression. These results were supported by several studies that identified other pathways involved in the regulation of autophagy in addition to the classical PI3K/AKT axis [43, 44].

In addition to AKT, ERK was reported to regulate autophagy in many cell types [27, 45]. Our results showed that PD98059 effectively blocked p-ERK1/2 protein expression and inhibited LC3II/I expression. Overexpression of SIRT1 alone activated p-ERK1/2; however, overexpression failed to reverse the inhibition of LC3II/I expression caused by PD98059. Therefore, we suggest that ERK signalling plays an important role in autophagy promoted by SIRT1 in human mildly degenerative NP cells.

## 5. Conclusions

The present study demonstrated that Pfirrmann grade III with a normal height of IVD is the key stage for biomolecular therapy, and the activity of autophagy in NP cells is the highest at this stage. Furthermore, SIRT1 inhibited apoptosis of mildly degenerative NP cells by promoting autophagy via the ERK signalling pathway. These findings will assist in the development of novel therapeutic approaches for DDD treatment.

## Data Availability

All the data used to support the findings of this study are included within the article.
